# Cardiovascular MRI in Detection and Measurement of Aortic Atheroma in Stroke/TIA patients

**DOI:** 10.4172/2329-6895.1000139

**Published:** 2013-11-01

**Authors:** Theodore Faber, Ashley Rippy, W Brian Hyslop, Alan Hinderliter, Souvik Sen

**Affiliations:** 1 University of South Carolina School of Medicine, Department of Neurology, Columbia, South Carolina, USA; 2 University of North Carolina School of Medicine, Department of Radiology and Cardiology, Chapel Hill, North Carolina, USA

**Keywords:** Cardiovascular MRI, Transesophogeal echocardiogram, Aortic atheroma

## Abstract

**Background:**

Aortic Atheroma (AoA) is an independent risk factor for new and recurrent stroke. AoA ulceration and mobility are associated with an increased risk for brain embolism. Transesophageal echocardiography (TEE) is the gold standard for detection and measurement of AoA in stroke/TIA patients. Cardiovascular MRI (cMRI) could be an alternative, non-invasive imaging modality for stroke/TIA patients. The objective of this study was to assess the accuracy and correlation of AoA detected and measured by cMRI versus TEE in patients with recent stroke/TIA.

**Methods and results:**

Twenty-two stroke/TIA patients undergoing TEE as a part of their stroke workup consented to a protocol-mandated cMRI performed on a 1.5 T magnet. The protocol included an axial non-breathhold EKG-gated dual-echo spin echo MRI of the thoracic aorta (TR/TE1/TE2=900/29/69) and a contrast-enhanced breathhold 3D gradient-echo image of the thorax (flip/TR/TE=12/4.0/1.71). Maximum plaque thickness, ulceration (≥ 2 mm) and mobility of AoA were assessed in the proximal (ascending and proximal arch) and distal (distal arch and descending) segments of thoracic aorta by a cardiologist to interpret the TEE and a radiologist to interpret the cMRI. There was good correlation between cMRI and TEE in measurement of plaque thickness in the proximal segments (R=0.73, p<0.0001) and the distal segments (R=0.81, p<0.0001) of the aortic arch (AA). cMRI had a high degree of accuracy in detecting measurable AoA (≥ 1 mm) in the proximal segments (sensitivity 90%, specificity 100%), as well as the distal segments (sensitivity 67%, specificity 100%). cMRI also had a high degree of accuracy in detecting significant AoA (≥ 4 mm) in proximal segments (sensitivity 71%, specificity 93%), as well as distal segments (sensitivity 71%, specificity 100%).

**Conclusion:**

The study showed a high degree of accuracy and correlation of AoA detected and measured by cMRI as compared to TEE in patients with recent stroke/TIA. This technique has limitations in detection of AoA ulceration, and protocols assessing AoA mobility need to be developed.

## Introduction

By any measure, the impact of stroke in the developed world is substantial. In the U.S., stroke is the fourth leading cause of death and a primary cause of significant disability [1]. It is essential to identify the was used to visualize the AA and identify aortic atheroma (AoA) in cryptogenic ischemic stroke and transient ischemic attack (TIA) patients. Correlation with TEE in 22 consecutive patients with cryptogenic ischemic stroke and TIA was made in order to assess source of stroke for appropriate treatment and secondary prevention [2]. Cryptogenic stroke, i.e. stroke without identifiable cause, may account for 30–40% of all stroke [3–6] and an increasingly recognized source of embolic stroke appears to be atheroma of the thoracic aorta, second only to atrial fibrillation in terms of risk [7–11]. Proximal aortic arch (PAA) atheroma plaques are seen in 16–20% of stroke/TIA patients [7]. Distal aortic arch (DAA) atheromatous plaque burden is a predictor of coronary disease [12], visceral thromboembolism, and mortality [13]. The disease burden in proximal DAA has also been demonstrated to be a frequent source of stroke via retrograde flow by Harloff et al. in 2010 [14]. Transesophageal echocardiography (TEE) is routinely requested in patients with cryptogenic stroke in order to identify a potential embolic source; however, it is moderately invasive and not tolerated by all patients. Moreover, the DAA is sometimes difficult to visualize due to airway obscuration [15]. Magnetic resonance imaging (MRI) is routinely used to investigate embolic potential from atheromatous disease in carotid arteries. This study proposes to analyze whether MRI can be useful to investigate embolic potential from proximal and distal aortic arch.

In this study, cardiovascular MRI (cMRI) of the aortic arch (AA) whether cMRI was able to identify AoA in these patients and to compare the characteristics of lesions identified by cMRI to those revealed by TEE.

## Methods and Materials

### Subjects

With institutional review board (UNC Biomedical IRB) approval, twenty-two ischemic stroke/TIA patients (mean age 68 years, 11 males, 17 white, 3 African-American and 2 others) undergoing TEE for assessment of possible cardiac source of embolism at UNC Chapel Hill served as subjects for the study. All patients experienced a cryptogenic stroke as defined by TOAST criteria [16]. To be included in the study, patients had to be age 18 or above, with stroke or TIA in the last 90 days, AA atheroma detected and measured on a clinically indicated TEE, and the ability to consent. Key exclusion criteria included the inability to cooperate with the performance of the MRI test due to size or claustrophobia, prior history of decreased renal function, and irregular heart rate that could limit cMRI. Patients were also excluded if they were unable to undergo MRI scans due to a history of implanted ferromagnetic material or devices.

### Transesophageal echocardiography (TEE)

Multiplanar TEE was performed on each patient. All patients received topical lidocaine pharyngeal anesthetic and adequate conscious sedation was induced by intravenous midazolam and fentanyl. The 5.0 MHz transducer of a Hewlett-Packard model 21364A transesophageal probe was used (Hewlett-Packard, Andover, Massachusetts). A Hewlett-Packard Sonos 1000 system was utilized and digital images were recorded for subsequent analysis. Imaging and quantification of AoA was conducted according to previously described methods [7]. In brief, the proximal and midascending aorta were imaged at a probe depth of 30 cm with a multiplane angle of 100° to 150° to view the vessel in the long axis. The descending thoracic aorta was examined by advancing the probe to the distal esophagus, imaging the aorta in cross section (at 0°), and then slowly withdrawing the probe to image the proximal segments. As the transducer reached the aortic arch plaque, the multiplane angle was rotated to between 0° and 90° to acquire sequential short-axis views. Digital images were acquired of the diseased areas in each segment of the aorta with annotation of the distance of the transducer from the incisors. None of these patients had fever or TEE evidence of valvular vegetation suggestive of infective endocarditis.

### Aortic Arch Plaque Thickness (AAPT) measurement

AAPT measurements are the maximal intima-media thickness (IMT) and grade of aortic atheroma [9]. In each patient the area of maximum aortic arch plaque was identified and imaged with Doppler turned off. The 2D images through the plaque were then obtained. A minimum of 2–3 images were captured and recorded for the arch segment of the aorta.

### Cardiovascular MRI (cMRI)

A 1.5 T magnet cMRI was used for all studies. The 3T magnet was used to optimize imaging and further improve resolution. An axial non-breathhold EKG-gated dual echo spin echo MRI of the thoracic aorta (TR/TE1/TE2=900/29/69) and a contrast enhanced breathhold 3D gradient-echo image of the thorax (flip/TR/TE=12/4.0/1.71) was performed on each patient. AA was assessed using spin-echo and 3D images. Plaque thickness was best assessed using the turbo spin-echo images to discriminate between the native aortic wall and plaque. The turbo spin-echo imaging had a repetition rate of three (3 averages). For larger areas of plaque that invaginated into the aortic lumen, contrast-enhanced imaging was employed. Plaque thickness was measured using measurements tools on the agfa PACS.

## Results

**Figure 1:**
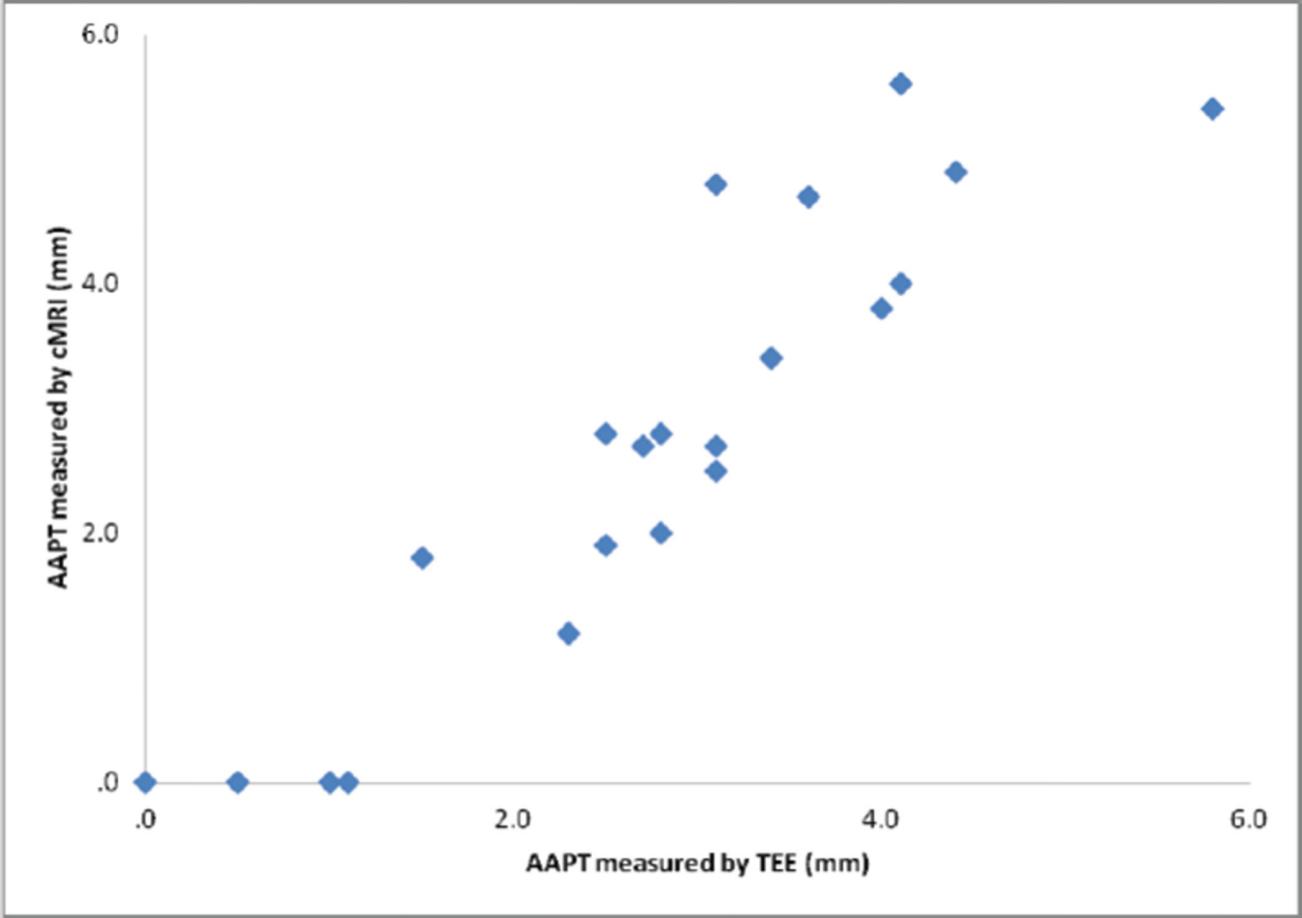
AAPT correlation in proximal segments.

**Figure 2:**
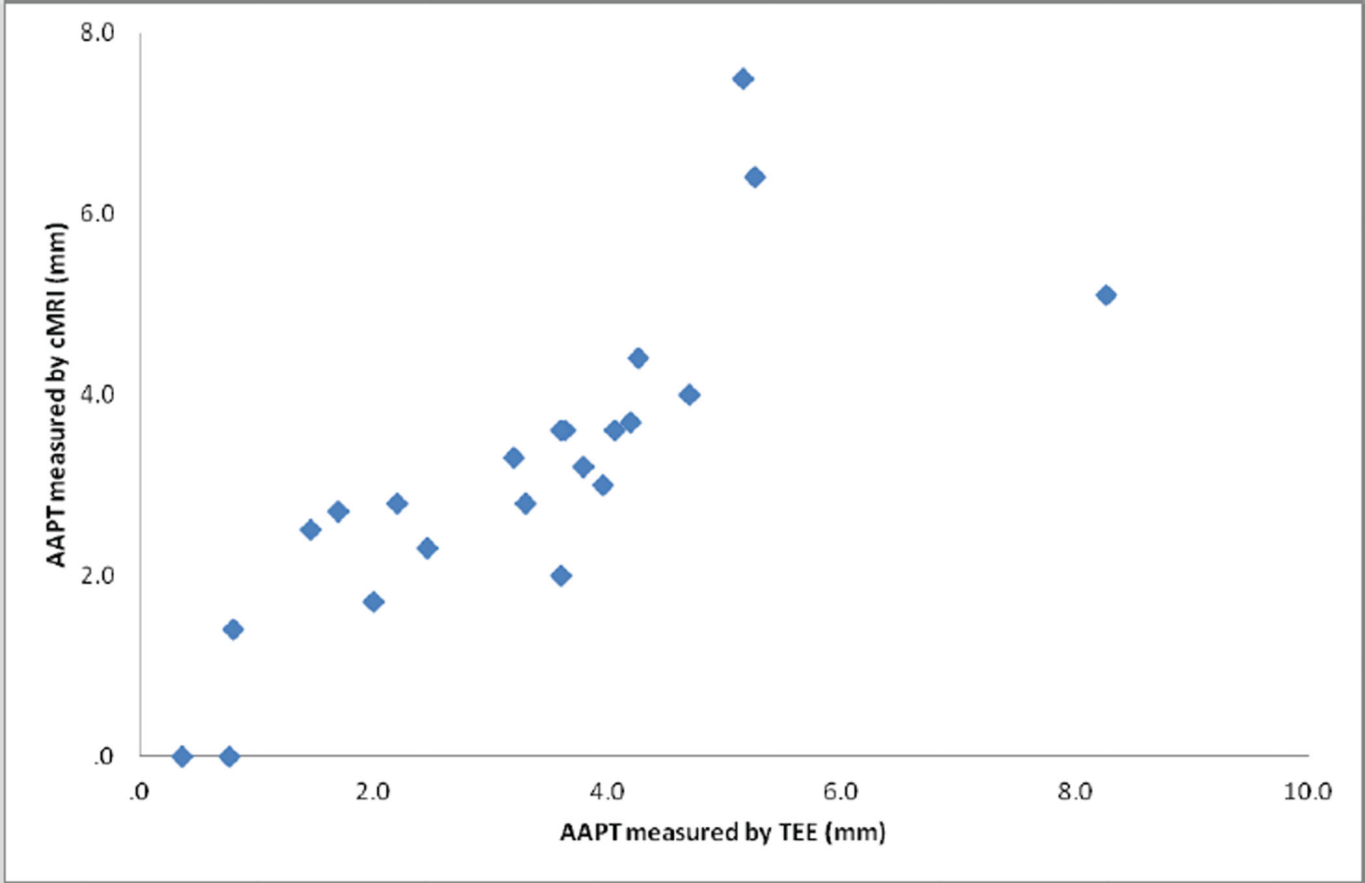
AAPT correlation in distal segments.

**Figure 3:**
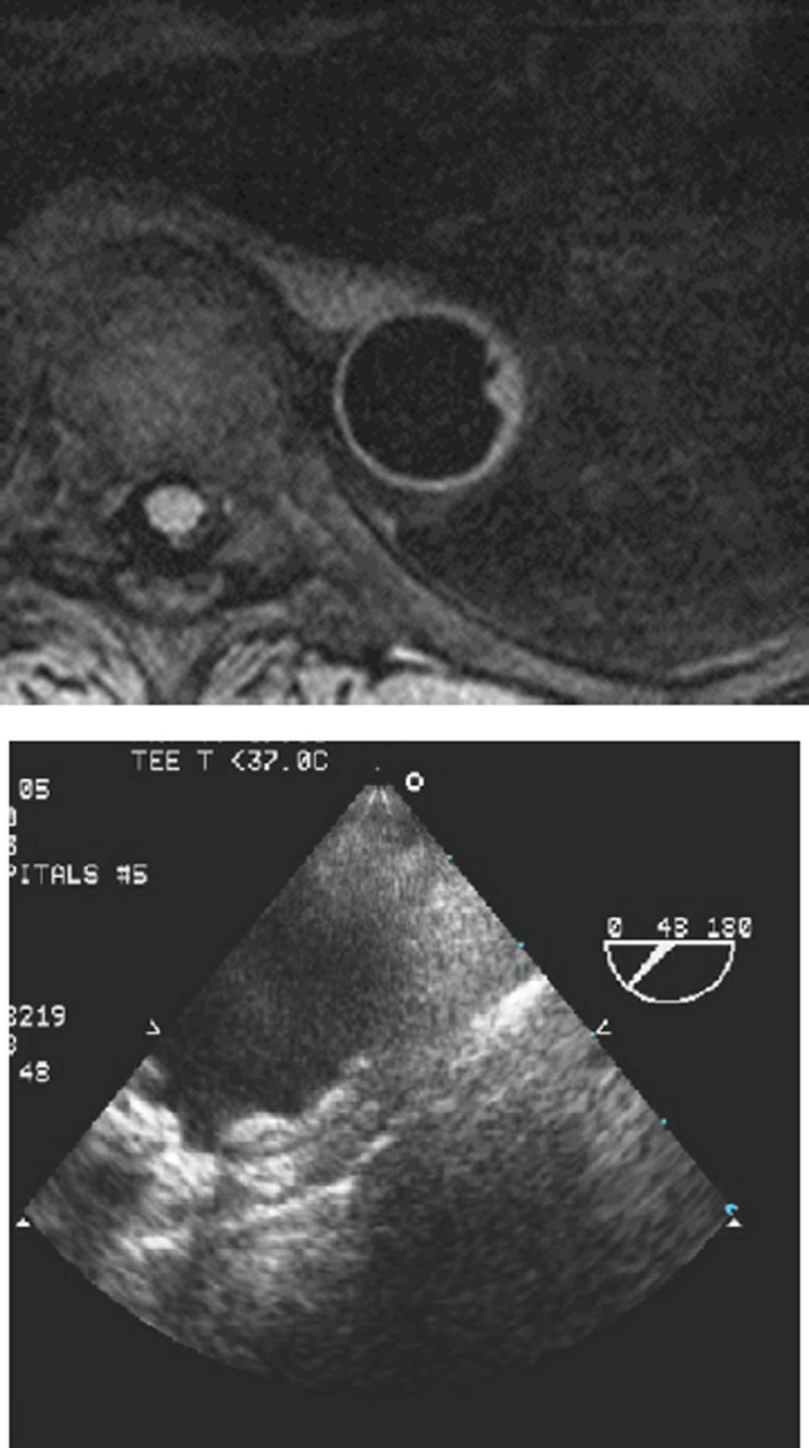
AA ulceration seen on cMRI and TEE.

There was good correlation between cMRI and TEE measurements of plaque thickness in both proximal segments (Pearson’s *R=0.726* and *p-value <0.001*) and distal segments (Pearson’s *R=0.812* and *p-value <0.001*) of the aortic arch (Figures 1 and 2). cMRI detected of proximal segment measurable AA disease ≥ 1 mm in thickness with 90% sensitivity and 100% specificity. In distal segments of the aortic arch, the sensitivity was 67% and specificity was 100%. cMRI detected severe AoA disease (≥ 4 mm in thickness) in proximal segments of the aortic arch with 71% sensitivity and 93% specificity. In the distal aortic arch, the technique was 71% specific and 100% sensitive. cMRI was able to identify 89% of proximal aorta ulcerations and 64% of distal aorta ulcerations (Figure 3). The mobility of plaques could not be assessed with this protocol.

## Discussion

It has been shown that aortic atheromatous (AoA) disease may be found in 16–20% of patients with transient ischemic attack (TIA) or stroke and a number of prospective studies have confirmed a strong association between stroke and AoA plaques [7,8,17–20]. Sen et al. [21] found a high rate of progression of aortic atheroma in 28% of ischemic stroke/TIA patients using transesophageal echocardiography (TEE), which is considered the gold standard for detection of AoA plaque in this setting [22]. TEE is widely available, produces images of greater resolution than transthoracic echocardiography (TTE) and has high inter-reader reproducibility [23,24]. However, TEE has significant limitations. It is semi-invasive, requires conscious sedation, and carries risk of damage to the esophagus, albeit low [24]. Patient tolerability may be a problem [25]; a relatively large number of individuals refused a follow-up TEE after a 12 month interval in one series [21]. Because of the semi-invasive nature of the study, patients for TEE should be hemodynamically stable. Many patients with stroke have difficulty in swallowing [26] and thus may have difficulty swallowing the transducer. Air artifact and insonation angles can interfere with visualization in proximal [15,24] and distal aortic arch [27]. A failure to reliably assess the aortic arch because of insufficient visualization was noted in one study in about half the patients tested; it was shown that TEE may underestimate AoA disease [27]. Difficulties with plaque thickness measurement and gradation scoring by TEE at precise locations in baseline and follow-up measurements was a limiting factor in a study of progression of AoA disease and risk of stroke [28].

Cardiovascular magnetic resonance imaging (cMRI) of the thoracic aorta has been shown to have unique characteristics which may improve the potential for identifying the source of cryptogenic stroke; about 21% of a group of patients with stroke of undetermined etiology were found to have AoA disease with embolic potential which was not detected by TEE [27]. The development of new protocols for cMRI to assess atheromatous disease of the ascending and descending aortic arch may help to reduce the numbers of cryptogenic stroke and to help understand the pathophysiology of AoA disease. Analysis of the morphology and potential stabilizing or destabilizing features related to the makeup of the plaque can be identified by cMRI, potentially leading to improved understanding of the processes by which AoA disease progresses [29–32]. Descending aortic arch AoA disease was not recognized as a potential source of stroke [33,34] until it was shown by Harloff et al. [14] using cMRI that retrograde flow with subsequent destabilization of complex atheromatous plaques in the proximal portions of DAA could potentially embolize to all supra-aortic arteries. This finding extended the risk of stroke by retrograde embolization to all brain territories and retina in about one-fourth (9/37) of serial unselected patients with cryptogenic stroke. MRI imaging of associated cardiac structures can be useful in detecting left ventricular thrombus [35–37], left atrial appendage thrombus [38], and patent foramen ovale, although evaluation of the latter requires contrast enhancement [39,40]. Reliable and accurate co-registration techniques utilizing cMRI may resolve precision issues [39]. Finally, cMRI may streamline ischemic stroke/TIA evaluation with concomitant imaging of the brain, cerebral and extracerebral vasculature, and cardiac/AA regions with a single examination which may impact overall costs associated with hospitalization [41].

MRI in general and cMRI specifically have limitations. The machines are not portable and represent a substantial investment on the part of the health care system. The presence of hardware including cardiac pacemakers, implanted defibrillators, and cochlear implants which might preclude cMRI is not uncommon in this patient population. Problems with patient size and potential for claustrophobia can be limiting factors. MRI is not performed in patients who are hemodynamically unstable, although this is also a limitation of TEE as noted above. EKG gating techniques may not be able to accommodate very irregular cardiac rhythms [41]. Portions of the cMRI sequence that require a breath-hold may not be feasible for some patients [41]. Finally, plaque mobility could not be assessed using the current protocol, although cine-cMRI [30] and other advanced techniques [42] may help overcome this limitation.

## Conclusion

This study demonstrates a high degree of accuracy and reliability of cMRI of the aortic arch in patients with cryptogenic ischemic stroke/TIA as compared to TEE. To our knowledge, this is the first and largest group of patients with cryptogenic stroke undergoing comparative evaluations of these two imaging techniques. Baher et al. confirmed the effectiveness of the ability of cMRI to reduce the rate of cryptogenic classification in a group of 15 patients (10/15 reclassified as cardioembolic) with cryptogenic stroke in 2012 [25]. Limitations of our study included the modest size of the study and lack of ability to visualize plaque mobility and limited ability to detect ulceration. Continued refinement of cMRI techniques is needed to overcome the technical limitations. Further investigation with greater numbers of subjects with longitudinal analyses of treatment is recommended in order to further understanding of the natural history of AoA disease and to determine optimal treatment paradigms in acute stroke and TIA as well as stroke prevention. Economic analysis of a streamlined MRI approach to the evaluation of cryptogenic ischemic stroke/TIA may be helpful considering the current concerns regarding healthcare expenditures.
